# Frailty links the heterogeneity of tinnitus disorder and response to interventions in older patients

**DOI:** 10.3389/fneur.2025.1617821

**Published:** 2025-08-12

**Authors:** Jian Ruan, Min Zhang, Zhao Han, Jie Chen, Qingwei Ruan, Zhijun Bao

**Affiliations:** ^1^Department of Otolaryngology, Huadong Hospital Affiliated with Fudan University, Shanghai, China; ^2^Shanghai Key Laboratory of Clinical Geriatrics, Shanghai Institute of Geriatrics and Gerontology, Huadong Hospital Affiliated with Shanghai Medical College, Fudan University, Shanghai, China; ^3^Department of Geriatrics, Huadong Hospital Affiliated with Fudan University, Shanghai, China; ^4^Laboratory of Aging, Anti-aging & Cognitive Performance, Shanghai Institute of Geriatrics and Gerontology, Huadong Hospital Affiliated with Fudan University, Shanghai, China

**Keywords:** tinnitus disorder, frailty, heterogeneity of tinnitus disorder, vulnerability, neural mechanism, functional connectivity, intervention

## Abstract

In addition to having a sensory component, tinnitus disorder might also be involved in tinnitus-related distress, cognitive dysfunction, and/or autonomic arousal, resulting in different behavioral changes and functional disabilities. The response to interventions has been shown to be heterogeneous in patients with tinnitus disorder. The underlying neural mechanisms of the heterogeneity of tinnitus disorder and their response to interventions remain elusive. Frailty and tinnitus share similar risk factors, including genetics; personality; early experience, aging and psychosocial stress; aging-related chronic systemic inflammation; vascular damage; neurodegenerative pathology; and functional decline in physical, cognitive, and psychosocial dimensions. The mechanisms by which frailty is linked to tinnitus disorder involve dysfunction of the HAP axis, cognitive and emotional processing, autonomic reactivity, and immune and metabolic regulation. Moreover, tinnitus, as a stressor, results in increased allostatic load, maladaptation, and adverse outcomes in individuals with frailty. The maladaptation induced by frailty contributes to the heterogeneity of tinnitus disorder, and the response to intervention is the synchronization of intrinsic brain networks characterized by increased integration and decreased segregation. Frailty may be associated with tinnitus chronification and relapse after effective intervention. We propose a model hypothesis to explain the bidirectional relationship between frailty and tinnitus disorder. In this model, the dysfunction of dynamic executive functioning might be the common pathway of tinnitus disorder and frailty. Investigating the efficacy of interventions for older people with frailty and tinnitus disorder will provide evidence about their benefits and disadvantages. Further exploration of vulnerability-related cortical and subcortical biomarkers of frailty and tinnitus disorder could provide guidance for the understanding and personalized prevention of tinnitus disorder.

## Introduction

Tinnitus, the conscious awareness of tonal or composite noise for which there is no identifiable corresponding external sound source, is a stress-related disorder (1, 2). The global prevalence of diagnosed tinnitus and chronic tinnitus is 3.4 and 9.8%, respectively, and that of severe tinnitus is 2.3%, ranging from 0.5 to 12.6% (3). The prevalence of tinnitus among adults is 14.4%, varies widely from 4.1 to 37.2%, and increases in prevalence with increasing age (9.7% among adults aged 18–44 years, 13.7% among those aged 45–64 years, and 23.6% among those aged ≥65 years) (3).

The majority of tinnitus sufferers can adapt and live in harmony with tinnitus. However, some individuals with tinnitus may experience adverse consequences, such as fear, annoyance, anxiety, depression (4–10), insomnia (11, 12), attention difficulty, cognitive impairment (13–15), and suicidal ideation (16, 17). Compared with those without tinnitus, tinnitus sufferers reported ~3 times higher prevalences of depression and anxiety (18). Improvements in depression and anxiety symptoms could decrease the prevalence of tinnitus (19). Depression and anxiety symptoms may precede tinnitus onset and increase the risk of tinnitus onset and evolution (20).

To differentiate it from tinnitus, which is a non-specific symptom of hearing disorders, DeRidder et al. (1) proposed that tinnitus with associated affective suffering be referred to as tinnitus disorder, which is chronic tinnitus (a duration of 3 months or more) associated with emotional and/or cognitive dysfunction and/or autonomic arousal, leading to behavioral changes and functional disability. The main symptoms and severity of tinnitus and tinnitus disorder are shown in Table 1. Unpleasant experiences of behavior and subjective feelings, such as negative cognitive, emotional, and autonomic impacts induced by tinnitus disorder, can lead to a lower quality of life and a greater cumulative societal cost (4). Tinnitus disorder is dynamic. In ~18% of patients with tinnitus, it resolves spontaneously, whereas in the other 82% of patients with tinnitus, it persists for 4 years on average (21). Among those with persistent tinnitus, tinnitus eventually improves in 9% of patients and worsens in the other 9%.

**Table 1:** Symptoms and severity of tinnitus and tinnitus disorder.

**Domains**	**Trigger/reaction**	**Symptom/distress**	**Severity**
Physical domains	Otological diseases	Noise hearing loss	Acute or chronic tinnitus: Only somatic symptoms without associated distress
		Age-related hearing loss	
		Meniere's disease	
		Ototoxic hearing loss	
		Otosclerosis	
		Middle ear infection	
	Non-otological diseases	Neurological diseases	
		Cardiometabolic diseases	
		Head trauma	
		Hypothyroidism or hyperthyroidism	
		Multimorbidity	
		Polypharmacy	
Affective domains	Persistently high level of tinnitus or other negative emotions about tinnitus	Despair	Tinnitus disorder: Mild: chronic tinnitus with only one of symptoms from affective, cognitive or autonomic arousal. Moderate: Chronic tinnitus with two symptoms specified in two domains from affective, cognitive or autonomic arousal. Severe: Chronic tinnitus with two or more symptoms specified in affective, cognitive or autonomic arousal, plus one very severe or multiple somatic symptoms
		Frustruction	
		Demoralization	
		Depression or anxiety	
		Annoyance, fear, worry	
		Irritability	
		Concentration difficulties	
		insecurity	
		Social withdrawal	
		Suicidal ideation	
Cognitive domains	Disproportionate and persist thoughts about tinnitus severity	Negative thinking	
		Attention	
		Memory	
		Language	
		Executive function	
Autonomic arousal	Excessive time and energy devoted to tinnitus	Insomnia	
		Vasoconstriction	
		Pain/headache	
		Increase of the nighttime systolic and diastolic blood pressure	
		Increase in the blood volume pumped by heart	
		Expanding the respiratory pathway	
		Dizziness	

Tinnitus disorder is heterogeneous and includes tinnitus triggers, perceptions, distress (the psychological reaction to tinnitus perception), and variations in treatment response (22). Vulnerability factors to tinnitus, such as personality traits, beliefs (23), chronic stress (24), cognitive reserve (15, 25, 26), and frailty (27), also contribute to tinnitus heterogeneity. Tinnitus triggers include aging, noise exposure, ototoxicity, and other comorbid medical conditions related to hearing or hidden hearing loss, emotional distress, attentional state, and somatosensory factors (28, 29). Tinnitus perception includes tinnitus pitch, e.g., noise or tone, tinnitus laterality, and tinnitus frequency. Tinnitus heterogeneity has resulted in the constant evolution of models of tinnitus pathophysiology in the literature. The cognitive–behavioral model (23), which evolved from the neurophysiological model (30) and habituation model (31), argues that tinnitus-induced negative thinking and behavioral changes create and maintain tinnitus distress. The tinnitus stress model indicates that chronic stress results in homeostatic imbalance, allostatic load, and maladaptation and supports a bidirectional connection of the auditory component and other components, such as emotional, cognitive, and arousal status (24, 32). The brain maladaptive plasticity model considers that maladaptive neural plasticity beginning at the cochlear nucleus causes increased spontaneous rates and synchrony in the central auditory system and extends to non-auditory structures or brain networks, generating different tinnitus perceptions and responses (28, 29). The integrative model of tinnitus involves the integration of the tinnitus perceptual core and other multiple parallel dynamically changing and partially overlapping subnetworks for tinnitus affective and cognitive components (33). This integrative model also integrates prediction error, deafferentation and thalamocortical dysrhythmia, noise cancellation, and a brain homeostatic model (33–35). Most of these models focus on the underlying brain mechanism of tinnitus and neglect the status of other physiological systems.

Frailty is defined as a decline in the functioning of multiple physiological systems, accompanied by compromised homeostasis and increased vulnerability to stressors, including physical, cognitive, and sociopsychological factors (36, 37). Like tinnitus disorders, frailty can occur at any age, but its prevalence increases with age (36, 37). Among community-dwelling adults, the prevalence of frailty ranges from 11% among those aged 50 to 59 years to 51% among those aged 90 years and over (38). Frailty is dynamic and fluctuates among different states of severity. Frailty phenotypes, such as physical frailty (36), cognitive frailty (39), social frailty (40), psychological frailty (41), and nutritional frailty (42), contribute to the vulnerability in different dimensions. Therefore, frailty might not only play an important role in the pathophysiology of tinnitus disorder, especially the non-auditory components, but also contribute to the heterogeneity of the response to tinnitus interventions.

In this study, we searched PubMed for articles published in English in the past 5 years, with the search terms “chronic tinnitus”, “tinnitus disorder”, “heterogeneity of tinnitus”, “response heterogeneity to tinnitus interventions”, “frailty”, “frailty and tinnitus”, and “neuroimaging of frailty or tinnitus”. We also sought publications from the reference lists of identified papers, including systematic reviews and clinical trials. We first reviewed the risk factors for frailty linked to the heterogeneity of tinnitus disorders and potential biological mechanisms. Next, we presented the heterogeneity of the response to tinnitus interventions, which might be linked to frailty. We reviewed the altered brain structures and functional connectivity (FC) of the neural networks associated with both tinnitus disorder and frailty and proposed a network model of tinnitus disorder associated with frailty, which might explain the heterogeneity of tinnitus disorder and the response to interventions. Finally, we presented the potential implications of integrating frailty into tinnitus disorder management for personalized interventions for tinnitus disorder.

## Frailty linked to the heterogeneity of tinnitus disorder

### Risk factors linking frailty and tinnitus disorder

A plethora of factors contributing to frailty are associated with the heterogeneity of tinnitus disorder. Genetic factors, early development, and early- and midlife adverse experiences, such as socioeconomic disadvantages, abuse, divorce, and job loss, contribute to age-related allostatic load increases, which are associated with frailty (43–45), pain (46), personality change (47), and chronic diseases, e.g., depression (48), cardiovascular disease (49), and hearing loss (50, 51). Allostatic load resulting in frailty and other health disorders might contribute to the auditory and non-auditory components of tinnitus disorder and the impairment of the physiological, cognitive–emotional, and behavioral response systems. Although there is no direct relationship between the allostatic load and tinnitus disorder, chronic stress, including genetic factors and genetic correlations with hearing loss and depression (52), socioeconomic status and long-term stress exposure (2, 9, 53–55), are directly related to tinnitus disorder.

Other demographic and social factors, such as advanced age, loneliness, maladaptive personality traits, and living alone, which increase the frailty risk, are also linked to age-related hearing loss and chronic tinnitus (41, 56, 57). Lifestyle factors, including diet, smoking, alcohol intake, sleep hygiene, and physical inactivity (58, 59), are associated with tinnitus disorder. Later-life risk factors for frailty, such as multimorbidity (4, 60), chronic diseases (61, 62), polypharmacy (63), malnutrition (64), micronutrient deficits (65, 66), depressive symptoms (5, 7, 10, 18, 19, 25), and cognitive impairment (13–15, 25, 26), are also linked to the onset and progression of tinnitus disorder. Among these risk factors, biological aging and psychosocial stress might be the main risk factors.

### The biological mechanisms of frailty associated with the heterogeneity of tinnitus disorder

**Figure 1:**
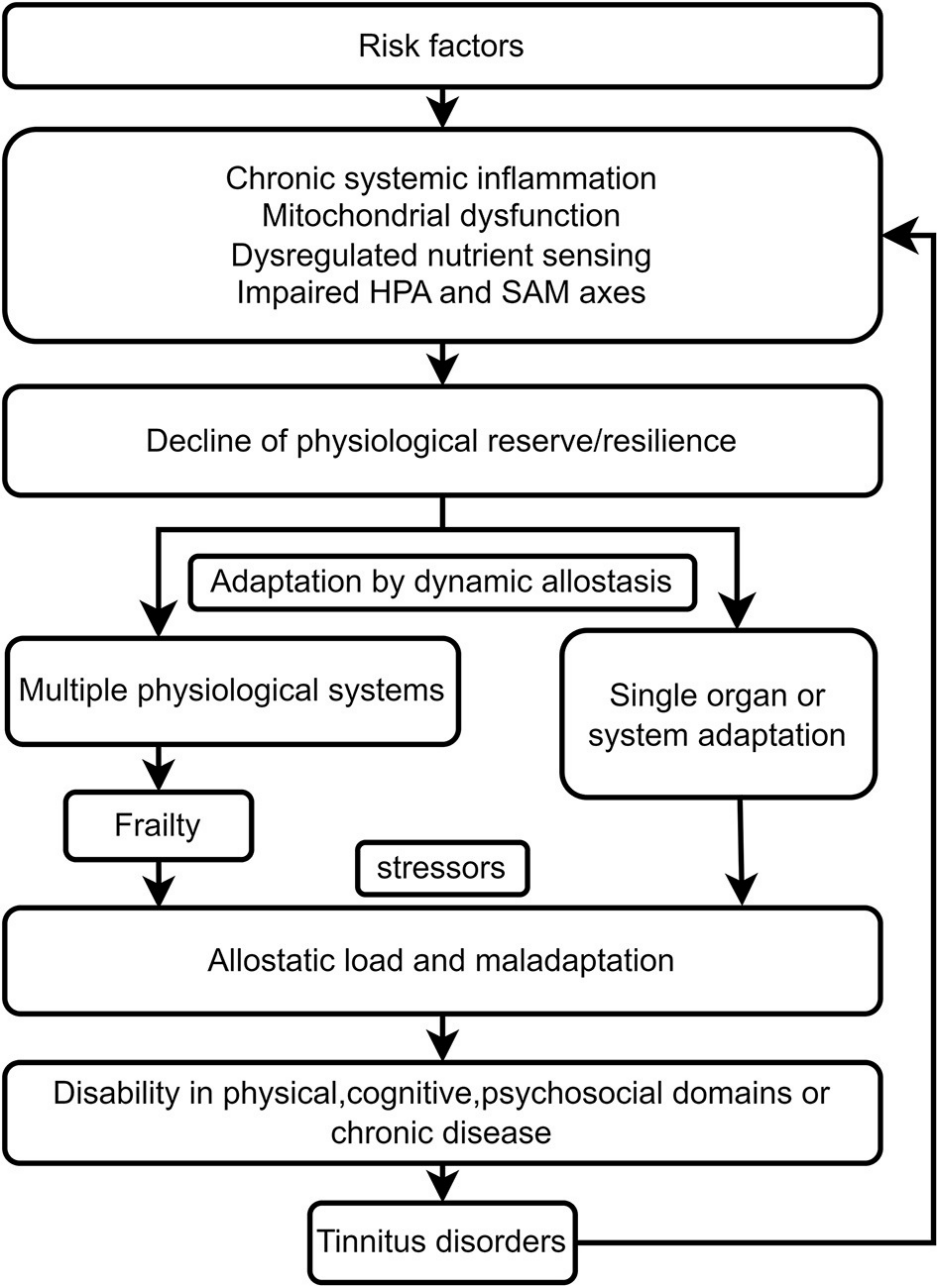
Frailty links tinnitus disorder resulting from age-related biological, environmental, and psychosocial factors.

Risk factors for frailty, including individual differences in vulnerability, such as genetics, early development, life-course experiences, personality traits, lifestyles and behaviors, and other physical, psychosocial and environmental challenges, interact and result in accelerated aging at the subcellular and cellular levels (37, 67). These include cellular senescence secretory phenotypes and mitochondrial dysfunction-induced oxidative stress and chronic systemic inflammation. Deregulated nutrient-sensing systems, including the insulin and IGF-1 signaling pathways for glucose sensing, the mammalian target of rapamycin (mTOR) pathway for the sensing of high amino acid levels, and AMP-activated protein kinase (AMPK) and sirtuins 1 and 3 for the sensing of low-energy states, also participate in the onset and development of frailty (37). Impaired hypothalamic–pituitary–adrenal (HPA) axis and sympathetic adrenal–medullary (SAM) axis function induce a decline in the response of the neuroendocrine, immune, and autonomic nervous systems to stress exposure (24, 67). Age-related hormonal changes, including dysregulation of thyroid, estrogenic, androgenic, growth and stress hormones also contribute to a decline in the reserve/resilience of multiple physiological systems (24, 37, 67). Complex physiological systems adapt to challenges or stress by dynamic allostasis. A decline in resilience and long-term adaptation results in frailty (Figure 1). Minor stressors, such as minor infection or surgery, might cause decompensation and maladaptation due to dysregulation of physiological systems and disproportionate changes in health status caused by allostatic load (24, 68). Different frailty phenotypes can lead to disability in different domains, including the physical, cognitive, and psychosocial domains. The vulnerability to different domains of frailty could explain why older subjects are more likely to present with disability, cognitive impairment, anxiety and depression associated with tinnitus than younger tinnitus patients do. The long-term failure of a single organ or system to adapt due to the allostatic load might cause pathological changes and chronic diseases, including hearing loss, chronic tinnitus and mood disorders (69). The pathological changes include neuronal atrophy, white matter lesions, demineralization, and dysfunction of the immune and metabolic systems.

The biological mechanisms of frailty onset and adverse outcomes also contribute to tinnitus disorder, including chronic inflammation and oxidative stress, mitochondrial dysfunction, dysregulation of the neuroendocrine and immune systems, HAP and SAM axes, and neurotransmitter activity (2, 24, 32, 70–72), are also involved in tinnitus. Tinnitus patients exhibit blunted reactive cortisol and dysregulation of negative feedback in the HPA axis (2, 24, 32, 73). Tinnitus can act as a stressor and results in adverse outcomes of frailty by inducing dysregulation of the HAP and SAM axes, including anxiety, depression, and sleep disturbances. Neuromodulation by stimulation of the vagal nerve or the auricular branch of the vagal nerve can alleviate not only neuroinflammation (72) but also tinnitus-related stress through improvements in parasympathetic activity and the balance of the autonomous nervous system (74). Peripheral inflammatory markers, such as significantly lower C-reactive protein (CRP) (75) and IL-10 levels (76) and higher oxidative stress indices and total oxidant statuses (77), have been reported in tinnitus patients than in healthy controls. Animal experiments have shown that sirtuin 3 can enhance the mitochondrial glutathione antioxidant defense system and prevent age-related hearing loss by sensing low-energy states (78).

Tinnitus disorder can interact bidirectionally with frailty. Tinnitus disorder might be aggravated by frailty, and tinnitus disorder induced by the aforementioned risk factors might lead to an allostatic load and maladaptation in older frail individuals. Tinnitus symptoms might be a stressor, accelerate the presence of adverse outcomes from frailty in different domains, increase the transition from tinnitus to tinnitus disorder, and increase the heterogeneity of tinnitus disorder (Figure 1). Therefore, frailty might cause heterogeneity in tinnitus disorder, and tinnitus or tinnitus disorder could become a stressor and lead to adverse outcomes in frail older individuals, including the worsening of tinnitus disorder.

## Frailty links the heterogeneity of tinnitus disorder response to interventions

The targets of interventions for tinnitus disorder include the auditory percept and non-auditory components and the cognitive, emotional, autonomic, and behavioral reactions to tinnitus sounds. Specific medicines for tinnitus are lacking, but some antidepressants, antianxiety drugs and mood stabilizers might be effective for treating its comorbid psychological symptoms (79). Tinnitus intervention strategies in clinical practice include tinnitus retraining therapy (tinnitus-specific educational counseling and sound therapy), sound masking therapy and sound enrichment, psychological therapies, neuromodulation by transcranial current/magnetic stimulation or vagal nerve stimulation, amplification devices such as hearing aids or cochlear implants, and combinations of different interventions (80, 81).

For tinnitus disorder, targeting the auditory percept (frequency, loudness, laterality, or duration) may not be effective. Moreover, the perceptual characteristics of tinnitus do not appear to be correlated with non-auditory symptoms, quality of life, sleep or tinnitus handicap (82). Psychological therapies including cognitive behavioral therapy (CBT), acceptance and commitment therapy (ACT), and mindfulness-based interventions, such as cognitive therapy and mindfulness-based stress reduction (MBSR), have been introduced for non-auditory symptoms and quality of life of patients with tinnitus disorder. Among the above intervention strategies, strong evidence from numerous clinical randomized controlled trials suggests that CBT is an effective treatment for non-auditory components or tinnitus distress by improving anxiety, health-related quality of life, and negatively biased interpretations of tinnitus, although an absence of evidence for long-term effects of more than 12 months is lacking (81, 83–85). CBT reduces tinnitus distress by changing the way people think about and behave in response to tinnitus. Its efficacy is not affected by incorporating both ACT and MBSR into CBT or by how it is administered, such as face-to-face or online (86). However, ~35 to 45% of individuals with tinnitus disorder do not respond to CBT (86, 87). Different components of CBT can be used to address different symptoms. Behavioral amotivation due to dysfunction of the reward system is related to greater responses to behavior therapy, such as goal setting, and fewer responses to cognitive restructuring (88). Some clinical symptoms or signs reflecting specific vulnerabilities and the severity of tinnitus disorder, such as discomfort and anxiety levels, in patients with tinnitus could be considered predictors of response to CBT (89).

For the emerging repetitive transcranial magnetic stimulation (rTMS), only 23% of individuals with chronic tinnitus respond (90). Among patients with chronic tinnitus, compared with non-responders, responders to rTMS had higher baseline scores on the tinnitus questionnaire (90). Directly finding vulnerable subnetworks by resting-state functional MRI could predict whether tinnitus patients will respond to particular treatment types (91). Tinnitus patients with greater functional network connections in the salience network–right frontoparietal network were sensitive to rTMS, and the optimal intervention for patients with lower functional network connections in the auditory network–salience network and auditory network–cerebellar network was sound therapy utilizing tailor-made notch music training.

The heterogeneity of tinnitus disorder responses to interventions might involve specific alterations in physiological reserves in multiple systems, especially the vulnerability of neural networks or subnetworks. Thus, the non-auditory components of tinnitus may affected by frailty, including physical, cognitive, and psychosocial dimensions/phenotypes, due to the vulnerability of multiple physiological systems.

## Frailty links tinnitus disorder via the vulnerability of neural networks

The segregation and integration of brain networks contributes to normal brain function. Aging results in decreased segregation or reduced FC within subnetworks, including the default mode network (DMN), salience, executive control and sensorimotor networks, and increased integration or increased FC between subnetworks (91–94). The adaptive changes in FC function during aging may serve as a compensatory mechanism to maintain function in the body and brain organ systems due to atrophy of the cerebral cortex, especially temporal and prefrontal cortices, and white matter lesions (95, 96). Tinnitus disorder and frailty are age-related diseases. Decreased segregation and increased integration in several cardinal networks induced by aging results in dysfunction of dynamic executive function to internal or external stressors may be the common pathways.

### Tinnitus disorder and the vulnerability of neural networks

Tinnitus disorder involves phantom percepts and accompanying psychopathological reactions due to the abnormal function of auditory and non-auditory networks (28, 29, 33, 34, 97). Tinnitus is processed by three anatomically separable but interacting pathways, including lateral and medial ascending pathways and descending noise-canceling pathways (98). The bottom-up hyperactivity of auditory pathways is the critical trigger factor of tinnitus, and the medial tinnitus pathway, overlapping with the salience network, contributes to the cognitive, psychological, and behavioral responses of patients with tinnitus disorder. The medial and lateral pathways are separable and are commonly balanced by the top-down noise-canceling pathway, and induce auditory sensation Tinnitus perception and the transition from tinnitus percept to tinnitus disorder depend on further processing of auditory stimulation by the abnormal synchronization of the central executive control network, the salience network, and the DMN. Therefore, some individuals may have tinnitus without suffering, and other individuals may suffer without tinnitus. Hearing loss in some individuals cannot induce tinnitus percept but leads to adverse structural changes in the medial ascending pathways, such as atrophy of the auditory cortex, frontotemporal regions, cingulate cortex, insula, and amygdala (56). The vulnerability of auditory and cognitive control and emotion processing circuits causes dysfunctional activation, including decreased cognitive reserve for other executive functions, due to increased support of the cognitive control network to effortful listening, abnormal emotion regulation and reactivity (56). Hearing loss also contributes to behavioral responses, including social isolation and loneliness, which aggravate dysfunction in cognitive and psychological domains. Thus, individuals with hearing loss without tinnitus may experience similar disorders as individuals with tinnitus disorder, such as cognitive, emotional, and behavioral reactions.

The ascending lateral sound pathway encodes the auditory component of tinnitus. Auditory nerve fiber deafferentation (reduced FC within auditory network) results in maladaptive structural and functional plasticity, including homeostatic downregulation of tonic inhibition and reorganization of the cortical tonotopic map in the central auditory pathway, beginning in the dorsal cochlear nucleus (DCN), then in the inferior colliculus (IC), and finally in the auditory thalamocortical system (28, 29). Somatosensory and auditory afferent projections could be integrated into the fusiform cells of the DCN, which is correlated with the development of somatosensory tinnitus (28). Frequency-specific increases in spontaneous firing rates, abnormal neural synchrony, and burst firing in the DCN, IC and medial geniculate body (MGB) lead to abnormal theta-range resonant interactions between the thalamus and cortex, referred to as thalamocortical dysrhythmia, and tinnitus generation (28, 29, 33). Thalamocortical dysrhythmia is characterized by maladaptive changes in the auditory cortex resulting from deafferentation. The auditory cortex can obtain missing information from neighboring cortical cells through a selective increase in cortical excitability due to imbalanced neuronal excitation and inhibition or by dendritic and axonal rewiring. If the bandwidth of deafferentation is large, alternatively, the auditory cortex could pull the missing auditory information from parahippocampal memory (33, 35).

The ascending non-specific medial “suffering” pathway encodes affective components of pain, tinnitus, and other pathologies (56). Amygdala–thalamic reticular nucleus (TRN) circuit is the projection of the basolateral amygdala of the limbic system to the TRN (99, 100). The amygdala regulates TRN gating auditory information by excitatory projections from the basolateral amygdala to the TRN (101, 102). The cortex and thalamus simultaneously send excitatory collaterals to the TRN (103). Thus, the amygdala–TRN functions as an ascending gatekeeper, regulating the affective value from the medial pathway. The mediodorsal and ventromedial posterior nuclei of the thalamus relay the ascending auditory information to the rostrodorsal anterior cingulate cortex, anterior insula, and auditory cortex, which are correlated with tinnitus suffering (33, 98, 104).

The top-down noise-canceling pathway separately regulates abnormal auditory activity in the auditory thalamus from the lateral and medial pathways. The inhibition deficiency of the descending noise cancellation pathway plays a critical role in the generation of tinnitus in individuals without initial tinnitus triggers or deafferentation and without map reorganization (105). The noise-canceling pathway contains a frontostriatal top-down gating system circuits, which is related to the affective value of internal and external percepts (34, 99). The substrates of the frontostriatal gating system involve the ventromedial prefrontal cortex (vmPFC) and the nucleus accumbens (NAcc) of the basal ganglion and the subgenual cingulate, which are closely related to the generation and maintenance of tinnitus (106, 107). The projections of the vmPFC and limbic structure of the NAcc extend to TRN, a region that consists of a layer of inhibitory GABAergic neurons present between the thalamus and neocortex and produces a direct inhibitory input on the neurons of the sensory thalamocortical relay neurons (108). The significance of the salience of the acoustic signals due to abnormal TRN gating sensory information is evaluated by the circuits of the vmPFC, limbic structure of the NAcc and auditory cortex. The vmPFC does not properly suppress tinnitus-related hyperactivity in the thalamus, and aberrant neuronal excitability in the NAcc results in tinnitus-related distress (106).

However, the abnormal auditory signals induced by the interaction between the ascending and descending pathways do not yield tinnitus percepts, including tinnitus loudness and lateralization, tinnitus duration, and tinnitus suffering. The signal must be processed in multiple parallel, dynamically changing, and partially overlapping non-auditory networks with specific spontaneous oscillatory frequencies (35). Tinnitus loudness percepts require synchronized activation of the salience network, including the anterior cingulate cortex (ACC) and anterior insular cortex, via the lateral pathway and transmit signals into the awareness network or perception network, which includes the subgenual ACC, dACC, pACC, precuneus, frontal cortex, and parietal cortex (34, 35, 98, 109). Two subpathways of the lateral pathway, the tonotopic lemniscal and the less tonotopic extralemniscal ascending auditory pathways, can induce the tone and noise types, respectively (33). The signal from the medial and noise canceling pathways must be processed by the salience network and distress network (including the subgenual and dorsal ACC, anterior insula, and amygdala), which results in tinnitus suffering (106, 109). The dysfunctional plasticity in these circuits may be critical for the tinnitus percept and percept-induced distress response.

Synchronized activation of the executive control network, including the dorsal ACC, dorsolateral prefrontal cortex, and inferior parietal lobule, is involved in cognitive and behavioral impairments, such as negative automatic thoughts and selective attention to tinnitus (22). Thus, communication between the executive control network and distress network leads to worsening tinnitus suffering. The default mode network, including the medial prefrontal cortex, posterior cingulate cortex, and precuneus, which controls self-representational processing, could result in tinnitus chronification by becoming tinnitus and tinnitus suffering as an integral part of the self, the new normal default state when pathologically communicating with tinnitus-provoking networks (98, 110–112). The imbalance of the positive reward system, in which the main hub is the NAcc, and the negative reward system, in which the main hub is the lateral habenula, receives dopaminergic projections from the ventral tegmental area and influences reward functions, including valuation, decision-making, and learning, and behavioral changes, such as pain, anhedonia, and motivational disturbances (104, 113). The functional connectivity between the pregenual anterior cingulate cortex and auditory cortex with the NAcc induced by tinnitus might also be involved in tinnitus chronification (104, 114). Changes in connectivity between the lateral pathway and motor network are associated with physical disability. Nonetheless, the difference between underlying tinnitus and tinnitus disorder needs further investigation. The dysfunctional plasticity in each network could explain the heterogeneity of tinnitus disorder and the response to interventions.

### Frailty and the vulnerability of neural networks

Frailty is a major modifiable factor of biological age and an extreme aging status. Individuals with frailty show specific changes in the brain microstructure and FC. Gray atrophy and white matter lesions are associated with physical frailty (115, 116). Microstructural neuroimaging of those with physical frailty has revealed gray atrophy in the medial frontal cortex; the basal ganglia (BG) region, including the putamen, caudate, and thalamus; the anterior cingulate cortex; and white matter lesions in the body of the corpus callosum (117). Brain structural markers of cognitive frailty, such as frontotemporal and subcortical atrophy, increased white matter hyperintensities, and decreased white matter microstructure integrity, have been indicated in previous studies (118, 119), which is different from the characteristic medial temporal lobe atrophy in early Alzheimer's disease (120). These brain structural degenerations result in decreased segregation within subnetworks and increased integration between subnetworks, including the goal-oriented executive control netework, DMN, salience network, motor network, and reward network.

Physically frail individuals present reduced FC (synchronization) in fronto-partietal areas (the executive control netework, for maintaining and processing information in working memory, problem-solving and decision making) (121) and supplementary motor areas associated with motor function and reduced intranetwork FC within the fronto-partietal, ventral attentional, and posterior DMN (122). In an Irish longitudinal study, frailty, assessed by the frailty index (FI), a self-reported multidimensional deficit, could capture physical but not cognitive impairment. Connectome-based predictive modeling results indicated a positive correlation between the FC of the visual network and FI and a negative correlation between FC in the BG and FI (123). The highest node of both networks was the caudate, but with different FC patterns: from the caudate to the visual network, and from the caudate to the DMN-related areas, respectively. The different connectivity patterns along with FI could reflect different recruitments in the brain network to maintain daily physical performance (123). Patients with mild cognitive impairment and frailty (cognitive frailty), which were assessed by the multidimensional frailty index, indicated that increased FC between the right hippocampus and clusters in the temporal gyrus was positively associated with higher frailty index scores (124). Late-life depression (LLD) is usually combined with physical frailty, referred to as psychological frailty (41). Individuals with LLD exhibit aberrant FC within and between the salience network, the DMN, the executive control network, and the frontal striatal reward network (125, 126). Network-level disruptions in connectivity result in cognitive deficits with diminished top-down control salience of negative stimuli, negative thoughts and emotions, and motivational disturbance, and in turn, maladaptive behavioral manifestations.

### Frailty hypothesis of tinnitus disorder

**Figure 2:**
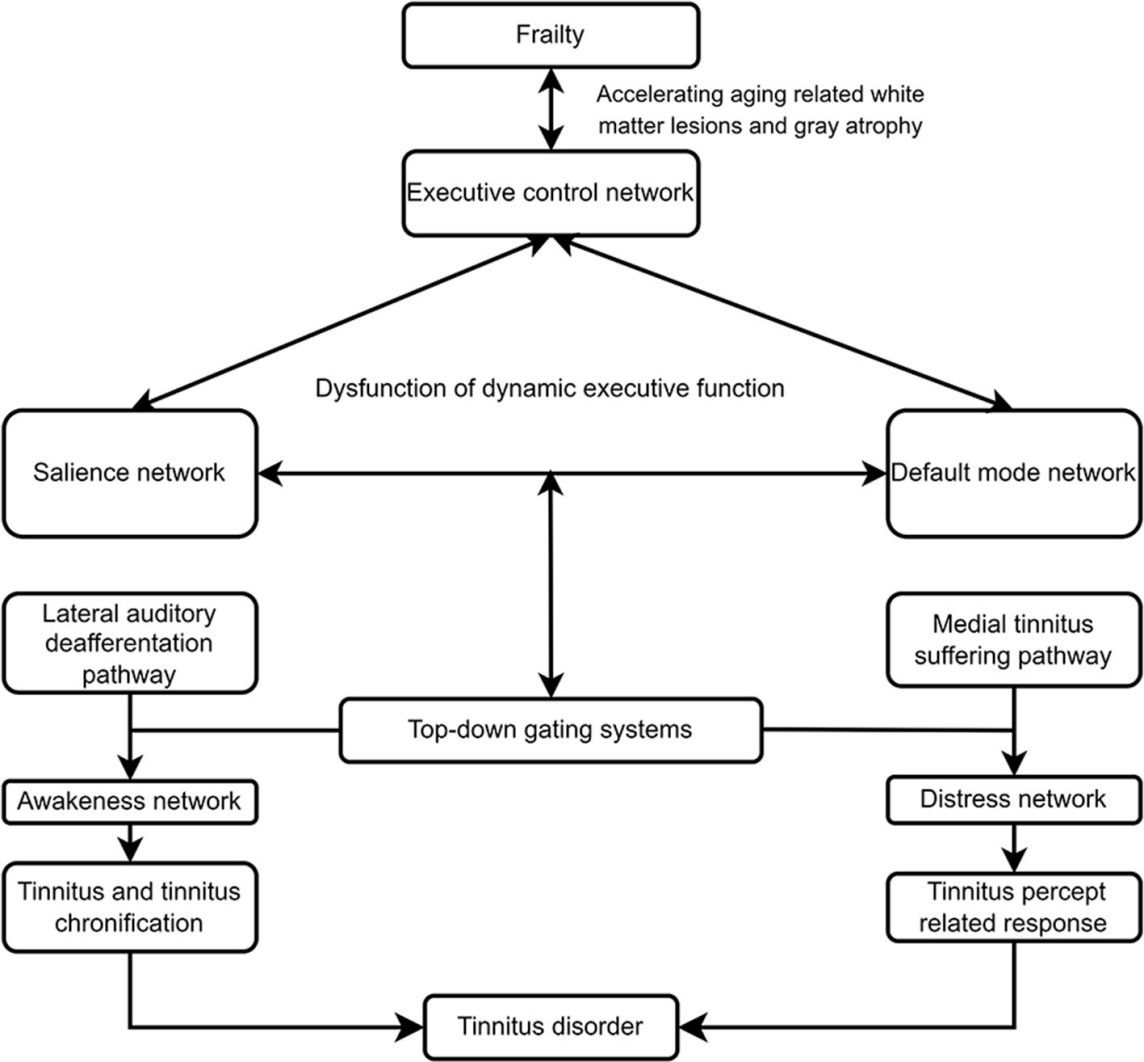
Frailly hypothesis of tinnitus disorder.

Tinnitus or tinnitus disorder likely interacts with frailty to produce ‘vicious cycling', which contributes to the heterogeneity of tinnitus disorder and the response to interventions. The hypothesized frailty model of tinnitus disorder revealed a bidirectional relationship between frailty and tinnitus disorder (Figure 2). Frailty accelerates biological aging and increases the vulnerability to morbidity in multiple physical, cognitive, and psychological dimensions. Frailty is associated with tinnitus severity (27, 127), cognitive impairment (128), depression (129), and other physical diseases. The risk factors for frailty, such as chronic inflammation, cerebrovascular pathology, and pathological protein burden in the brain, can cause white lesions in the brain (115–117), cortical and subcortical atrophy (118–120), and homeostatic imbalances in intrinsic functional networks (121–126). The intrinsic neural networks, such as the salience network, DMN, executive control network, motor network, and reward system, are disrupted to varying extents in patients with different frailty phenotypes or dimensions. The decreased FC intranetwork (segregation) and increased FC internetwork (integration) among the salience network, DMN, executive control network had been proposed to underline somatic symptom and neuropsychiatric disoders, including tinnitus, evidently contribute to the heterogeneity of disorders and responses to tinnitus interventions (Figure 2). Frailty results in dysfunction in the salience network, DMN, executive control network, and the interaction of these networks, including increase salience of negative stimuli and negative self-referential thinking, decreased cognitive control and selective attention, and the salience network driven switch between DMN and executive control network. Dynamic executive functioning hypothesis, referred to as a balance between the salience and the executive control networks had been proposed for individual to dynamically and quickly adjust the ongoing behavior when faced with a stressful event (130). Frailty decreases cognitive reserve and more cognitive effort demand for auditory function in older adult with tinnitus. The reallocation of cognitive resource might not meet the salience network to identify tinnitus and initiate the switch between DMN and executive control network, which lead to failure in tinnitus inhibitory control, and dynamic transition of tinnitus percept to tinnitus disorder, tinnitus percept and suffering chronification.

Tinnitus or tinnitus disorder may also accelerate frailty development and progression and contribute to the adverse outcomes of frailty (Figure 2). Typically, tinnitus percept or suffering, as a stressor, can result in the adaptation of neural and physiological responses by dynamic allostasis. Segregation and integration in the brain network could maintain homeostasis. However, repeated or continuous tinnitus, together with other risk factors, may cause frailty or the vulnerability of multiple physiological systems, including neural networks for cognitive and emotion processing. The sensation information of tinnitus percept from the ascending lateral auditory and suffering information from medial suffering regulated by top-down-noise canceling pathway activates salience, percept network, DMN, and executive control network by dynamic allostasis compensatory mechanism to trigger goal-oriented behaviors, including decreasing the salience of tinnitus percept and suffering, negative automatic thoughts of tinnitus. Moreover, the adaptive behaviors will be added to the repertoire of successful strategies for the response to the following same tinnitus percept or suffering and saving cognitive resources. In combination with aging and other factors, such as psychological stress, the vulnerability of dynamic executive functioning to maintain homeostasis will increase, resulting in tinnitus percept and/or suffering, the transition from tinnitus percept to tinnitus disorder, and frailty. furthermore, the interaction of tinnitus and frailty might cause allostatic load and maladaptation and adverse physical, cognitive, and psychological outcomes and chronic morbidity.

## Clinical implications: targeting frailty for tinnitus disorder

Both frailty and tinnitus disorder involve the functional impairment of physical, cognitive, psychological and behavioral alterations. The common vulnerability factors of frailty also correlate with the heterogeneity of tinnitus disorder, including relapse after remission following interventions, tinnitus chronification, increased comorbidity, and the heterogeneity of the response to tinnitus interventions by challenging functional networks and homeostasis in multiple physiological systems (Table 2). Individuals with the personality phenotype of neuroticism are associated with chronic diseases, AD, depression, and tinnitus distress through frequent dysregulation of autonomic reactivity and the HPA axis for homeostasis maintenance (57, 131, 132). Vulnerability factors in the physical domain are associated mainly with dysfunctions in sensory function, mobility, and balance in tinnitus disorder. Patients with physical frailty present with subclinical cardiovascular (133) and cerebrovascular damage (134), cortical infarcts, and reduced brain volume (115). Brain pathology, including AD pathology, macroinfarcts, Lewy body pathology, and nigral neuronal loss, which are associated with the rapid progression of frailty (135), might also be involved in the heterogeneity of tinnitus disorder and the response to interventions. Vulnerability factors in the psychosocial domain, such as negative beliefs and loneliness, are associated with the risk of depression, AD pathology, and a decline in physical and cognitive function due to the abnormal activation of the HPA axis and the overproduction of cortisol (136, 137). Negative beliefs are also related to tinnitus distress through the induction of negative autonomic thoughts about tinnitus (23). Individuals with an external locus of control have passive coping strategies, such as seeking help for tinnitus, and experience more tinnitus distress due to maladaptation to stressors (138, 139).

**Table 2:** Target frailty vulnerability factors related to tinnitus disorder.

**Domain**	**Vulnerability factors of frailty**	**Tinnitus disorder correlates**	**Pathological and functional correlates**
Personality	Neuroticism	Tinnitus distress, including cognitive impairment, depression, anxiety, and autonomic arousal	Elevated autonomic reactivity Dysregulated in HPA axis
Physical domain	Impaired sensory function	Hearing and vision loss, tinnitus, and tinnitus disorder, hearing related dysfunction in cognitive and psychological domain	Atrophy of auditory cortex, frontotemporal regions, atrophy and dysfunctional activation of ACC, amygdala, insula. Dysfunction of the cognitive control network. Malplasiticity in auditory pathway, increased FC between inter-networks.
	Sedentary lifestyle	Decline in mobility and balance function	Chronic systemic inflammation, Dysregulated in HPA axis, elevated autonomic arousal, and circadian processes. Muscle atrophy, cerebrovascular impairment, and neurodegeneration. Reduced FC of fronto-partietal areas, and supplementary motor areas, ventral attentional, posterior DMN, and increased FC from the caudate to visual network, and decreased FC from the caudate to DMN-related areas.
	Multimorbidity, chronic diseases, and polypharmacy	Dysfunction in mobility, balance, and tinnitus distress	
	Malnutrition (low protein intake and micronutrient deficits, vitamin B6, D and E)	Dysfunction in mobility, balance, and tinnitus distress	
	Disrupted or irregular sleep	Tinnitus distress	
Cognitive domain	Subjective cognitive decline and MCI (including preclinical-AD and early of vascular dementia)	Tinnitus distress, including negative thinking of tinnitus, selective attention	White matter lesions, atrophy in frontal brain, temporal gyrus, posterior parietal cortices, and hippocampus AD pathology Low FC in the executive control network, DMN, and memory areas.
	PD-like symptom	Dysfunction in cognition, mobility, and balance	Lewy-bodies and neuronal loss in the substantia nigra, white matter lesions, atrophy in cortices and subcotices, diminished functional connectivity of the supplementary motor areas.
Psychosocial domain	Internalized self-blame or stigma in beliefs	Tinnitus distress: Fearful beliefs, external locus of control, negative thought, distorted perception	Dysregulated in HPA axis, elevated autonomic arousal. White matter lesions, atrophy in temporal lobe, AD pathology. Low FC in the executive control network, DMN, salience network, and fronto-subcortical regions.
	Avoidance or passive coping	Intensifying negative thought, and tinnitus distress	
	Late-life depression symptom	Comorbid or tinnitus related depression	
	Anxiety symptom	Comorbid or tinnitus related depression	
	Social role absences, social withdrawal, loneliness	Dysfunction in physical, cognitive, and psychological domain	

AD, Alzheimer's disease; DMN, the default mode network; FC, functional connectivity; HPA axis, hypothalamic–pituitary–adrenal axis; MCI, mild cognitive impairment; PD, Parkinson disease.

The vulnerability factors of different domains of frailty result in FC vulnerability within specific subnetworks and internetworks, which, in turn, contributes to the heterogeneity of tinnitus disorders and the response to interventions. However, different frailty instruments, such as the Fried physical frailty phenotype with five criteria and frailty indices with different numbers of deficits, including signs, symptoms, disabilities, diseases and laboratory parameters, are used to assess frailty (36, 37), which limits the ability to find specific cortical biomarker signatures in the early stage of frailty. The BG, which includes the striatum, globus pallidus, thalamus, subthalamus, and substantia nigra, along with several subcortical brain structures, is critical for motor control and learning, cognitive function, and reward. Selective BG vulnerability has been observed in frail patients, as assessed by physical phenotype and FI (117, 123). The sensorimotor area, associative area, and limbic area of the BG lack anastomosis between the second- and third-order branches and collateral supply. The BG exhibits selective vulnerability to the risk factors for frailty and ischemic lesions due to energy deprivation (140). Dysfunction of energy metabolism is one of five criteria of the physical frailty phenotype and has been proposed as the basal component of prefrailty to construct a frailty phenotype and FI (141). Identifying characteristic cortical or subcortical biomarkers of prefrailty and different frailty phenotypes or dimensions will facilitate the personalized management of tinnitus disorder.

Frailty may influence the heterogeneity of tinnitus disorder and the response to tinnitus interventions. However, almost all clinical trials assessing the efficacy of various interventions for tinnitus disorder have not assessed frailty status. The conclusions of these studies might provide misleading information to clinicians. The inclusion of frail patients with tinnitus disorder in trial designs could provide evidence not only for the personalized treatment of tinnitus but also for understanding the vulnerability factors to predict the efficacy and response to interventions.

## Conclusion

The relationship between frailty and tinnitus is bidirectional with positive feedback. Risk factors cause a decline in multiple physiological reserves and vulnerability to tinnitus symptoms through chronic inflammation, vascular damage, neurodegeneration, and an impaired HPA axis. The adaptation of organs or systems by dynamic allostasis and the negative effects on dynamic executive functioning induced by vulnerability factors of frailty might contribute to the heterogeneity of tinnitus disorder and treatment responses. To implement patient-centered assessment of frailty domains and personalized care plan for older people with tinnitus disorder in clinical practice might be a feasible strategy to target the heterogeneity of tinnitus disorder and response to interventions. Understanding the specific mechanism and exploring characteristic cortical biomarkers of different frailty domains could further provide guideline of personalized treatment approaches and predict the efficacy of these approachesfor older people with tinnitus disorder.
